# *Notes from the Field*: Postexposure Prophylaxis for Rabies After Consumption of a Prepackaged Salad Containing a Bat Carcass — Florida, 2017

**DOI:** 10.15585/mmwr.mm6642a7

**Published:** 2017-10-27

**Authors:** Vikram Krishnasamy, Matthew R. Mauldin, Matthew E. Wise, Ryan Wallace, Laura Whitlock, Colin Basler, Clint Morgan, Dana Grissom, Sherry Worley, Danielle Stanek, Jamie DeMent, Pamela Yager, William Carson, Rene E. Condori, Yoshinori Nakazawa, Claire Walker, Yu Li, Christopher Wynens, Allison Wellman, James Ellison, Emily Pieracci

**Affiliations:** ^1^Epidemic Intelligence Service, Division of Scientific Education and Professional Development, Center for Surveillance, Epidemiology, and Laboratory Services, CDC; ^2^Division of Foodborne, Waterborne, and Environmental Diseases, National Center for Emerging and Zoonotic Infectious Diseases, CDC; ^3^Division of High Consequence Pathogens and Pathology, National Center for Emerging and Zoonotic Infectious Diseases, CDC; ^4^Florida Department of Health in Santa Rosa County; ^5^Florida Department of Health; ^6^Food and Drug Administration.

On April 3, 2017, two Florida residents consumed part of the same prepackaged salad before reportedly discovering the partial remains of a bat carcass in the salad. Bats are known reservoirs for rabies virus, which causes rabies disease in both animals and humans (*1*). The persons who ate the salad contacted the Florida Department of Health (FLDOH), which notified CDC’s Poxvirus and Rabies Branch. CDC and FLDOH determined that the immediate concern was for potential rabies virus exposure, because approximately 6% of bats submitted to U.S. public health departments annually test positive for rabies virus (*2*,*3*).

Although percutaneous exposures are more likely to result in successful transmission of rabies virus to humans (*1*), transmission can occur when infectious material, such as saliva or nervous tissue from an infected animal, comes into direct contact with human mucosa (*2*). Infection with rabies virus causes an acute, progressive encephalitis that is nearly always fatal once clinical signs have begun. The disease is preventable if exposed persons receive timely postexposure prophylaxis (PEP), which includes human rabies immunoglobulin and 4 doses of inactivated rabies vaccine administered over 14 days (*4*).

FLDOH submitted the bat carcass to CDC for rabies virus testing on April 4. Polymerase chain reaction and direct fluorescent antibody tests were inconclusive because of the deteriorated condition of the carcass. However, because the cranium of the bat was intact, exposure to brain material by the persons who consumed the salad was unlikely, although exposure to the bat’s organs or peripheral nervous tissue was possible. PEP was recommended because laboratory test results were inconclusive and exposure to nervous tissue could not be ruled out.

The salad was purchased from a company A store location. After being notified of the investigation, company A removed the lot of prepackaged salad from all store locations on April 5. Company B (the prepackaged salad supplier) recalled the affected lot of salads on April 8. CDC advised consumers to contact their local health department for PEP evaluation only if the consumer had eaten a recalled prepackaged salad and had found animal material in the salad. CDC was not notified of any other reports of dead bats in prepackaged salads.

To identify where the bat might have been introduced into the prepackaged salad, CDC performed genetic analyses on the bat to determine its subspecies. Based on morphology and phylogenetic analyses (Bayesian inference and haplotype network analyses) of mitochondrial DNA sequence data (Cytb and D-loop), the bat was identified as a Mexican free-tailed bat (*Tadarida brasiliensis mexicana*), which is found throughout the southwestern United States. It is genetically distinct from *T. brasiliensis cynocephala*, which occurs in the southeastern United States (Figure) (*5*).

**FIGURE:**
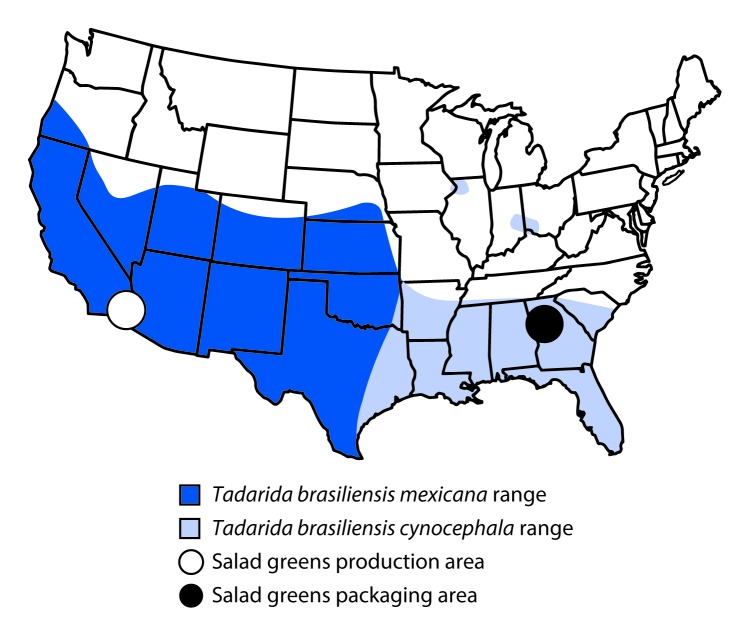
Distribution of *Tadarida brasiliensis mexicana* and *T. brasiliensis cynocephala* bat species in areas of production and packaging of salad greens — United States, 2017

The investigation determined that cutting and harvesting of greens for the recalled salad occurred in fields in the west and southwest United States before they were transported to a processing plant in Georgia. At the processing plant, the greens were washed with chlorinated water and packaged. Given the physical condition of the bat (e.g., decomposed, bisected) and the geographic location of the fields and the processing plant, along with the genetic identification of the bat, investigators concluded the bat most likely came into contact with the salad material in the field during harvesting and cutting and was then transported to the processing facility.

Several factors likely reduced the risk for rabies virus transmission to the two Florida consumers. No rabies virus was detected in the specimen, the bat’s cranium was intact, and the salad was rinsed before packaging, thereby diluting any potential virus. In addition, mucosal membrane exposures have rarely been proven to result in rabies disease, and rabies virus does not survive more than a few days outside a host (*2*). Although this exposure was likely of low risk, this investigation was an example of effective industry and government collaboration to remove a product of concern from the marketplace rapidly to protect consumers.
